# "Are you available for the next 18 months?" - methods and aims of a longitudinal birth cohort study investigating a universal developmental surveillance program: the ‘Watch Me Grow’ study

**DOI:** 10.1186/1471-2431-14-234

**Published:** 2014-09-22

**Authors:** Valsamma Eapen, Susan Woolfenden, Katrina Williams, Bin Jalaludin, Cheryl Dissanayake, Emma L Axelsson, Elisabeth Murphy, John Eastwood, Joseph Descallar, Deborah Beasley, Rudi Črnčec, Katherine Short, Natalie Silove, Stewart Einfeld, Margot Prior

**Affiliations:** Academic Unit of Child Psychiatry South Western Sydney Local Health District (AUCS), Sydney, Australia; School of Psychiatry and Ingham Institute, University of New South Wales, Sydney, Australia; Ingham Institute for Applied Medical Research, Sydney, Australia; Sydney Children’s Hospitals Network, Sydney, Australia; University of New South Wales, School of Women’s and Children’s Health, Sydney, Australia; Department of Paediatrics, University of Melbourne, Melbourne, Australia; Developmental Medicine, Royal Children’s Hospital, Melbourne, Australia; Murdoch Childrens Research Institute, Melbourne, Australia; Centre for Research, Evidence Management and Surveillance, Sydney and South Western Sydney Local Health Districts, Sydney, Australia; School of Public Health and Community Medicine, University of New South Wales, Sydney, Australia; Olga Tennison Autism Research Centre, La Trobe University, Melbourne, Australia; NSW Kids and Families (NSW Health), Sydney, Australia; Community Paediatrics, South Western Sydney Local Health District, Sydney, Australia; South Western Sydney Clinical School, South Western Sydney Local Health District, University of New South Wales, Sydney, Australia; Speech Pathology Unit, Liverpool Hospital, Liverpool, Australia; Discipline of Paediatrics and Child Health, University of Sydney, Sydney, Australia; Centre for Disability Research and Policy, Brain & Mind Research Institute, University of Sydney, Sydney, Australia; School of Psychological Sciences, University of Melbourne, Melbourne, Australia

**Keywords:** Child Development Disorders, Surveillance, Screening, Children, Preschool

## Abstract

**Background:**

Universal developmental surveillance programs aimed at early identification and targeted early intervention significantly improve short- and long-term outcomes in children at risk of developmental disorders. However, a significant challenge remains in providing sufficiently rigorous research and robust evidence to inform policy and service delivery. This paper describes the methods of the ‘Watch Me Grow’ study that aims to maximise accurate early detection of children with developmental disorders through a partnership formed between policy makers, service providers and researchers.

**Methods/Design:**

A mixed methods study design was developed consisting of: (1) a qualitative study of parents and health service providers to investigate barriers and enablers of developmental surveillance; (2) recruitment of a birth cohort and their longitudinal follow-up to 18 months of age to: a) assess risk factors for not accessing existing developmental surveillance programs and b) estimate the prevalence of children identified with developmental risk; (3) comparison of surveillance outcomes with a reference standard at 18 months of age to assess the diagnostic test accuracy of existing and alternative developmental surveillance tools; and (4) comparison of developmental surveillance models to inform policy recommendations. Data linkage will be used to determine the uptake and representativeness of the study participant group versus non-participants.

**Discussion:**

The Watch Me Grow study is expected to provide a collaborative opportunity to enhance universal developmental surveillance for early accurate identification of developmental risk. This will also provide quality evidence about identification of developmental risk and access to services to be embedded in existing practice with linkages to policy development.

## Background

### Importance of Universal Surveillance

There is increasing evidence that early detection and intervention for developmental disorders has the potential to alter adverse development and provide significant short- and long-term benefits to human capacity. These benefits include increased school retention and reduced unemployment [1–3]. Unfortunately, the majority of developmental difficulties are not detected until children start school [3]. The Australian National Health and Medical Research Council [4] and the American Academy of Paediatrics (AAP) [5] recommend a system of universal developmental surveillance where the risk of significant developmental problems, and the need for further assessment and early intervention, can be identified early. Developmental surveillance is a continuous and cumulative process whereby knowledgeable healthcare professionals identify children who may have developmental problems [5]. The key components of developmental surveillance include eliciting and attending to parents’ concerns about their child’s development; documenting and maintaining a developmental history; making accurate observations of the child; identifying risk and protective factors; and maintaining an accurate record of findings. It is critical that there is ongoing contact with families and children coupled with anticipatory guidance and promotion of child development within families as well as responding to developmental concerns reported by parents, followed by clinical observation and the use of a validated surveillance tool over multiple time periods [4, 5].

### What is currently known about Universal Surveillance methods?

Reviews of current practice in primary healthcare and anecdotal Australian evidence suggest that there is inconsistency in how developmental surveillance is undertaken in primary healthcare [6, 7]. Studies have documented the difficulties with approaches to monitoring developmental progress in child health settings [8], which typically involve parents/carers raising concerns during a consultation, and/or the administration of screening tools. While there are benefits of surveillance, there are also barriers to developmental surveillance achieving its potential positive impact. These include time constraints and difficulties in accessing high quality and affordable primary healthcare for children according to need [9], and obstacles for children receiving appropriate interventions even when they are recognised as being at risk of developmental delay [10–13]. For example, in Australia there are long waiting periods for both private and public assessment and intervention services for identified developmental problems such as speech and language disorders or global developmental delay [14]. Moreover, the lack of data regarding the uptake of the developmental surveillance program and service utilisation creates challenges for policy makers, service providers, and clinicians in developing appropriate care pathways; a key issue that the 'Watch Me Grow' (WMG) study seeks to address [15].

We are not aware of any robust, longitudinal evidence on the uptake of universal developmental surveillance in communities, particularly those with high levels of socioeconomic disadvantage. Additional gaps in existing evidence include the nature of the barriers and enablers to the uptake of developmental surveillance by families and the accuracy of developmental surveillance in identifying children at risk of developmental disorders such as autism spectrum disorder (ASD) [16, 17] and intellectual disability. Finally, there is scant data regarding models of partnership between policy makers and service providers to meet the challenges in delivering universal or targeted interventions for those at risk.

### Developmental Surveillance in New South Wales

In 2007, the New South Wales (NSW) Ministry of Health introduced the Parents’ Evaluation of Developmental Status (PEDS) [18] for routine administration as a surveillance tool to be completed by child health professionals such as a Child and Family Health Nurse (what we will refer to as a community nurse in this paper) or by general practitioners (GPs). The PEDS is a 10-item standardised parent-report questionnaire that systematically elicits parental concerns regarding their child’s health, development, and behaviour to estimate that child’s developmental risk [5, 19]. The PEDS has moderate reported sensitivity of 74–80%, and specificity of 70–80% in validation studies from the USA [20], however to our knowledge no diagnostic test accuracy study has yet been conducted within Australia, notwithstanding a small study examining the PEDS’ capacity to detect symptoms of ASD [21]. The PEDS is designed for completion by parents/carers of children from birth to 7 years and 11 months of age. It takes about two minutes to administer. Of the ten questions, eight cover expressive and receptive language, fine motor, gross motor, behaviour, socialisation, self-care, and learning while the other two are about more general learning, development, and behaviour. Parents can respond ‘yes’, ‘a little’, or ‘no’. Two or more predictive concerns places a child at high developmental risk; one predictive concern places a child at moderate developmental risk, and one or more non-predictive concerns or no concerns places a child at low or no risk [22]. In NSW, the Department of Health recommends that children at high and moderate developmental risk are further assessed by a child health professional using a secondary developmental screening tool, the Ages and Stages Questionnaire (ASQ) [23] and Ages and Stages Questionnaire: Social-Emotional (ASQ:SE) [24].

In NSW, the PEDS is included in the child’s Personal Health Record (PHR; commonly known as the ‘Blue Book’), which is given to all parents at their child’s birth, and is to be completed at regular check-ups (at 6 months, 12 months, 18 months, 2 years, 3 years and 4 years). In Australia, developmental surveillance varies among states and territories. The PEDS is now the first line developmental surveillance tool used in many states and territories of Australia, including NSW, Victoria, Tasmania, the Australian Capital Territory, Western Australia and urban areas of the Northern Territory. In South Australia, the ASQ is used for developmental surveillance. The ASQ is also used in NSW and Western Australia as the second tier developmental surveillance tool as described above, while in Victoria and the Northern Territory the Brigance Screens [25] are used to follow-up developmental risk detected by the PEDS. In Queensland, community nurses conduct developmental reviews, but standardised tools are not used. In addition, the Queensland primary care program, which is run by nurses, targets families who are identified as high risk, mainly through the child’s first two years of life.

There are also significant between- and within-state differences with regard to pathways to diagnostic assessment following identification of children at developmental risk. For example, in NSW, the subsequent assessment depends on the pathway developed by areas within local health districts, and can include referral to a paediatrician, GP or a local child development clinic.

In addition to the issues inherent in the choice of tools, procedures and follow-up pathways, there are also significant barriers and enablers relating to health systems and policies as well as in parental behaviours that influence the uptake and participation in surveillance programs. Based on a national survey in the USA, the AAP reported that while most paediatricians agreed that developmental issues should be addressed, they were less confident about their ability to undertake this activity [26]. This survey identified a number of barriers for health professionals in completing developmental monitoring, including time constraints, inadequate reimbursement, lack of non-physician support staff, lack of further diagnostic and treatment services, insufficient training, and lack of familiarity with assessment tools. In Australia a survey of GPs in central and south western Sydney identified that less than half (44%) use the NSW Blue Book which includes the PEDS to discuss developmental concerns with parents [27]. At the same time, 60% of GPs who were surveyed reported that there were barriers to families seeking help for their children at risk of developmental disorders. These barriers included waiting times, cost, availability and access to services, and being from a non-English speaking background [27]. Using a case scenario method in which a developmental paediatrician considered a 2.5-year-old child as needing further follow-up, a study observed that one fifth of GPs responded that they would not initiate further assessment [27]. In this regard, the uptake of current developmental surveillance methods appears to be poor.

Unfortunately, families with the greatest needs for basic services such as food, housing and healthcare are often the least likely to receive support because of difficulties encountered in accessing these basic services [28]. These families are also likely to have children with a higher than average risk of having a developmental or behavioural difficulty. Furthermore, the predominant needs of children with developmental or behavioural problems including developmental assessment, speech and language therapy, and support for parents in managing challenging behaviours, are often limited in disadvantaged areas, making it difficult for families to access these services [29].

### Methodological considerations for the Watch Me Grow study

To date, studies providing evidence about surveillance programs have provided key pieces of information, but in a way that has been disconnected from other information that is required. This has been because study design was cross-sectional rather than longitudinal, only quantitative or qualitative rather than both, and often not embedded within a service system. These are key methodological limitations in the existing literature that form part of the rationale for the WMG study.

## Methods/Design

### Aims

Within the WMG study, our objectives are to:

1. examine barriers and enablers for universal developmental surveillance in NSW from the perspective of policies, systems and processes using focus groups and in-depth interviews of stakeholders.
2. assess risk factors for non-completion of 6-, 12-, and 18-month developmental surveillance from the perspective of parent participation and engagement;
3. determine the prevalence and psychosocial correlates of developmental risk at these ages; and
4. ascertain the diagnostic test accuracy of the current NSW universal developmental surveillance program and whether the addition of an autism specific screening tool at 18 months increases diagnostic accuracy.

The WMG study is a mixed methods study that includes both quantitative and qualitative components. The qualitative component is designed to evaluate the feasibility, and barriers and enablers of the current universal surveillance program in NSW (objective 1), whereas the quantitative component seeks to provide data addressing objectives 2, 3 and 4.

### Setting

This study is being conducted in south western Sydney, an area of significant social disadvantage about 40 km from the Sydney central business district. The South Western Sydney Local Health District (SWSLHD) provides tertiary (including neonatal intensive care, inpatient and outpatient paediatric care) and community health services to the residents of this region. Children are referred to the Sydney Children’s Hospital Network for subspecialist paediatric services. SWSLHD is the largest health service in NSW and comprises seven local government areas. In 2011, the population of SWSLHD was estimated at 875,384 persons (11.7% of the NSW population), and is projected to increase by 18,000 people per annum over the next decade. By 2016, the population is expected to reach 958,397 people and 1.06 million by 2021. There were 12,997 births in south western Sydney in 2012, representing about 13 per cent of all births in NSW [30]. The total fertility rate ranges from 1.90 to 2.34 infants per woman compared to the NSW rate of 1.97 infants per woman [31]. This is a fast growing population including a large indigenous and culturally and linguistically diverse (CALD) community, characterised as having high unemployment, and the accompanying health and psychosocial concerns of disadvantaged populations [31].

### Study design

#### Objective 1: qualitative study

Participants in this component of the study will comprise the main stakeholders, namely parents, community nurses, GPs, paediatricians, general practice nurses, pharmacy nurses, pre-school and day-care staff. We will conduct focus groups as well as in-depth individual interviews with participants to identify barriers to the universal developmental surveillance program, community’s awareness, accessibility and engagement with the surveillance program as well as the factors that might enable universal developmental surveillance. Parental knowledge about typical/atypical early childhood development and professional knowledge about developmental surveillance systems will also be ascertained. Participants from a range of socioeconomic and CALD backgrounds will be included in order to ensure that we fully capture the experiences including barriers and enablers to using the health services of families from a wide range of ethnic and language backgrounds. We will interview families from the main multicultural groups resident in south western Sydney, and use translations of study information and interpreters during this key stage.

The Grounded Theory Method will guide the interpretation and thematic analyses of these qualitative data as well as feed into hypotheses of later stages of the study [32].

#### Objectives 2 and 3: longitudinal study

**Figure 1:**
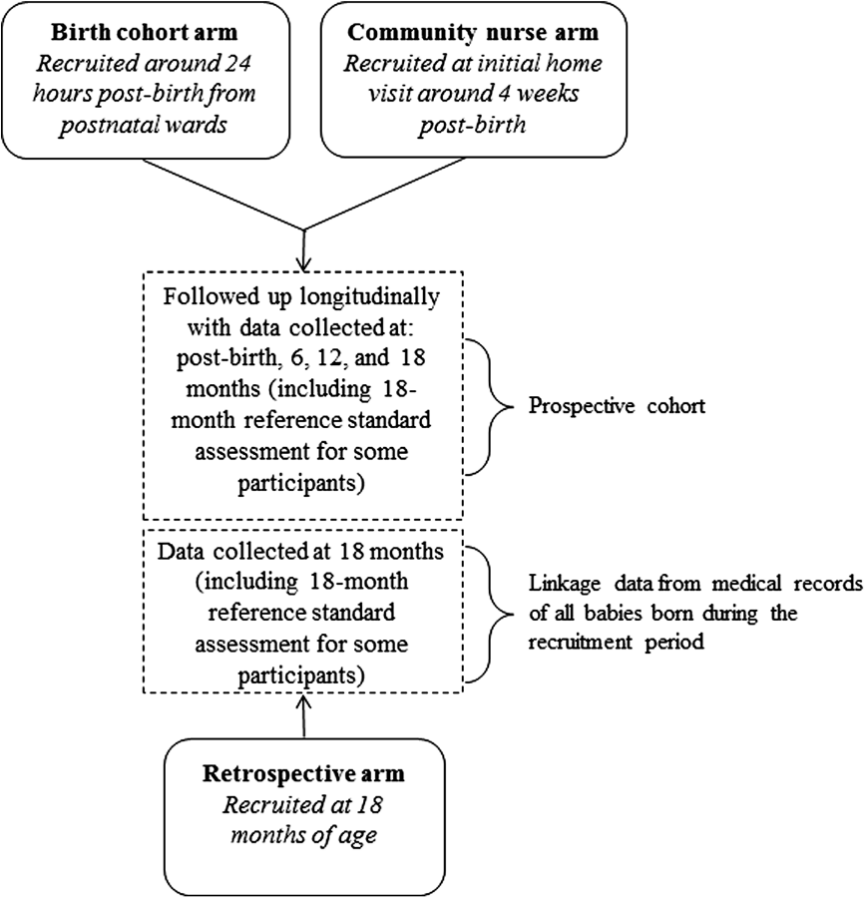
**Watch Me Grow study recruitment.**

A cohort of children will be recruited in three main study arms (see Figure 1). A birth cohort from two teaching hospitals in SWSLHD will form the primary target sample (birth arm). An additional prospective cohort will be recruited through community nurses in the community as this is the pathway being used currently in NSW for developmental surveillance (community nurse arm). These two study arms will be combined into the main prospective cohort of the study (prospective cohort). In addition, a retrospective arm of the study will recruit infants born in the two teaching hospitals that are currently in the age range of 18 to 21 months (retrospective cohort) and will serve as a comparison group.

For the birth arm, the WMG research team will attend the postnatal wards on a daily basis to recruit mothers who have given birth in the previous 24 hours at the two teaching hospitals in SWSLHD. The nursing staff on the postnatal wards will be consulted and their support sought for recruitment into the study. Recruitment information will be made available in English, Arabic, Vietnamese, Khmer, and Chinese - the predominant community languages used in south western Sydney. Families who agree to participate in the study will be asked to complete a Newborn Baseline Questionnaire (NBQ) which includes baseline sociodemographic and health service-use information, and will be informed about the subsequent follow-up telephone interviews when their infant is 6, 12, and 18 months of age.

For recruitment to the community nurse arm, parents will be informed about the study by community nurses during the Universal Health Home Visit that occurs by four weeks post-birth. If the family shows interest in learning more about the study, the contact information of parents who express interest will be provided to the WMG study research team. Researchers will then contact these parents and mail study information and consent forms with reply-paid envelopes to be returned.

The retrospective arm of the study will comprise infants born in the two teaching hospitals who are in the age range of 18 to 21 months at the time of recruitment. This sample of children will serve as a ‘surveillance as usual in the community’ comparison group (i.e., a control group). This will help to determine any participation bias by ensuring that the WMG study is not influencing the help seeking and health related behaviours of study participants via a Hawthorne effect. For example, it is possible that the awareness gained through participation in the study might make the families more compliant with the developmental surveillance programs being assessed.

The total target sample for the study from all three arms is expected to be 2000 children.

##### Follow-up of prospective cohort

Telephone follow-up interviews for the prospective cohort (i.e. those recruited through the birth and community nurse arms of the study) will consist of a 10-minute interview that will ask about their use of universal developmental screening services using a purposively developed Prospective Developmental Surveillance Questionnaire (PDSQ). These telephone interviews will occur after the infants reach 6, 12, and 18 months of age. The key questions will include whether the child has been taken for the recommended scheduled checks, which health service(s) has been used, satisfaction level with the service, whether a PEDS had been completed, and by whom. The researcher contacting the family for the follow-up calls will complete the PEDS with any parent who has not completed a PEDS as part their child’s personal health record developmental surveillance schedule. At the 18-month telephone call, components of the NBQ will be re-administered so that it is possible to compare the sociodemographic characteristics at the child’s birth and 18 months later, as well as further questions on social capital, access to early childhood education services, and parenting. The same procedures will be followed for the retrospective cohort, but all the data will be collected in one telephone call.

##### Underlying theoretical model and data analysis for longitudinal study

In order to plan comprehensive service models from the findings of the longitudinal study, we need to examine the complex transactional relationship between the child’s environment and biology over time [33]. We have developed a composite of a bio-ecological and life course model to serve as a framework within which to do this [34–37]. These theoretical models will be linked to appropriate analytical models and in this regard multilevel modelling will be employed to examine the independent impacts of community variables (e.g., socio-economic status), parental/family variables (e.g., family health, substance use, mental health history, country of birth, language spoken at home, parental perceptions about preventive healthcare and access to childcare and preschools), and child variables (e.g., temperament, preterm/low birth weight, intrauterine exposure to drugs, medicines, infections, low Apgar scores, perinatal complications, presence of developmental and behavioural concerns) on developmental risk and access to services. A combined risk index will be created that includes measures of biological, psychosocial, developmental and socioeconomic risk used in the previous multivariate analyses. Cumulative risk analysis will be undertaken to determine the relationship between burden of risk and uptake of referral recommendations and utilisation of services. In addition, we will examine the transactional impacts of these variables on the uptake of referral recommendations provided by the research team following the reference standard assessment and utilisation of health services.

#### Objective 4: diagnostic test accuracy study

All children who are identified as being at risk of having a developmental disorder using the PEDS (both ASQ positive and ASQ negative), will be invited to participate in a reference standard assessment (defined below) between 18 and 21 months of age. Of the approximately 2000 children that will be recruited to the study, it is estimated that a concern on the PEDS will be reported for around 800 children (40%) and of these approximately half (400 children) will be at a level of risk that warrants further assessment using the ASQ and the ASQ:SE. From the remaining 1200 PEDS negative children, every 12th child (*n* = 100) will also be invited to participate in all the additional assessments (ASQ, ASQ:SE, Modified Checklist for Autism in Toddlers, Mullen Scales of Early Learning, ADOS). Thus, it is planned that a total of 500 children will receive further reference standard assessments as described below.

The reference standard assessments will be conducted to assess whether the current program is accurately identifying children with ASD, global developmental delay, physical developmental problems, and speech and language concerns. We will also evaluate whether the addition of the Modified Checklist for Autism in Toddlers (M-CHAT) assessment [38] would improve correct identification of children with ASD. The parents will also complete the Short Temperament Scale for Toddlers [39]. The M-CHAT, ASQ, and ASQ:SE will be completed by the parents prior to the reference standard assessment but the researchers carrying out the assessments will be blind to the child’s scores on PEDS, M-CHAT, ASQ, and ASQ:SE.

Reference standard assessment tools: 1) Baseline assessment of developmental quotient will be made using the Mullen Scales of Early Learning [40], which is a standardised assessment of cognition from birth to 68 months. The scales show strong and continuous validity over time and across cultures and are widely used with pre-school children; 2) The Autism Diagnostic Observation Schedule 2nd edition–Toddler Module (ADOS) [41] is a semi-structured, standardised observational assessment of the child’s communication, social interaction, and play. This instrument has been designed to assist in the diagnosis of ASD, with a diagnostic algorithm generated that is consistent with the primary diagnostic classification systems. The instrument has excellent inter-rater agreement in diagnostic classification, good test-retest reliability and internal consistency. To ensure the reliability of the ADOS assessment, we will video all assessments and ten percent of the video samples will be randomly selected and reviewed by an independent assessor to assess inter-rater reliability. Once all the assessments are completed, this will be reviewed by a panel with clinical expertise in paediatrics, psychiatry, child and family health nursing and speech pathology as appropriate, to determine a clinical decision about the outcome of the assessments. Families of children identified to have features consistent with a developmental disorder will also be provided with information about the need for further clinical assessment. Service mapping and a local service directory of resources will be provided along with referrals to the local multidisciplinary assessment clinics covering the catchment areas of the two teaching hospitals.

Once these assessments are conducted we will calculate the test characteristics (sensitivity, specificity, positive and negative likelihood ratios) of 1) the PEDS alone, 2) the current NSW surveillance program (PEDS with second level ASQ); and 3) the PEDS plus M-CHAT by comparing the performance of these tools, alone or in combination, in correctly identifying children with a developmental disorder.

#### Power calculation

A sample size of 500 children is large compared to the majority of previous studies exploring test performance of developmental surveillance tools and combinations of tools. The PEDS has been reported to have a sensitivity and specificity of around 70 to 80% [20]. Assuming the prevalence of developmental problems in the population is 10%, a total sample size of 449 subjects will be required to achieve a confidence interval width of 12.5% (that is, precision) around a minimum sensitivity of 76% [42]. A smaller sample size will be required for a similar precision with a minimum specificity of 75% (*n* = 52). Allowing for a dropout rate of approximately 10%, a total of 500 subjects will provide sufficient experimental power.

#### Data quality checks

A large proportion of the NBQs will be completed by the parents at the postnatal wards at recruitment and the remainder, along with the PDSQs will be completed over the telephone. To ensure that the questions are answered in a format that is suitable for analysis and the researchers’ questionnaire completion is consistent, each questionnaire will be checked prior to data entry. Any missing or ambiguous answers will be re-asked at a subsequent telephone call or when researchers see the parents at the developmental assessments.

#### Data linkage

It is possible that we will miss some mothers in recruitment for reasons such as: delivery during weekends or public holidays, early discharge following delivery, and refusal to participate in the study. Thus it is important to ascertain the representativeness of the sample that will be recruited. This will be done through data linkage of our cohort with electronic medical records of all the mothers who delivered children during the recruitment period at the two hospitals. We will compare key socio-demographic factors such as age, education level, indigenous background, postcode of residence, country of birth, and languages spoken between the mothers recruited to the project and those who are not recruited.

Similarly, for the prospective cohort component, we will use data linkage to compare the rates of those who attend the 6-, 12-, and 18-month developmental checks from the study participant group with those who are not participants in the study. It is possible that women who agree to participate are more aware of and engaged with health services (including the surveillance program) as compared to those who are not participating in the study. Hence, relationships between various sociodemographic factors and attendance at a 6-, 12-, and 18-month health and/or developmental check with a healthcare professional will also be examined using data linkage with community nurse records. Similarly we will compare key sociodemographic factors for those who drop out of the study as compared to those who continue in the study until their infants reach 18 months of age.

#### Ethics

Ethical approval to perform the study has been obtained from the University of New South Wales Human Research Ethics Committee.

#### Promotion of the project

During the recruitment period, clinical and administrative staff will be informed about the study through clinical and research meetings as well as by displaying posters with information about the study on the wards and waiting rooms. A website has also been developed.

#### Management of project/governance

A Project Management Committee has been established with representatives from each of the partner organisations and quarterly meetings of this committee will provide the forum for strategic oversight, reporting and feedback. Further, a Research Implementation Committee has been formed to oversee the day-to-day operational aspects of the study. Monthly meetings will be held to discuss issues on an ongoing basis for each of the core components of the study, that is, qualitative focus groups, longitudinal follow-up, and 18-month diagnostic test accuracy components. Other stakeholders and national and international experts will be invited to participate in the policy translation phase.

## Discussion

There have been significant advances in developing effective and targeted interventions for developmental disorders in the last decade but a critical challenge remains in ensuring uptake of available developmental surveillance services, accurate detection, and timely referral of children at risk of, or with, identified problems to appropriate services. The research base on these issues is relatively limited, and often constrained by the use of cross-sectional rather than longitudinal approaches, as well as either qualitative or quantitative data without the combination of these methods. Moreover, difficulties in conducting research within ‘real-world’ health services means that samples are often drawn from university or other clinic settings. By seeking to address these methodological issues in the WMG study, our hope is to create, if you will, a three-dimensional picture of the issue and in so doing generate detailed possibilities for solutions.

While the developmental surveillance program rolled out in NSW using PEDS and ASQ is an excellent example of how early identification and follow-up pathways might operate, the uptake of the program appears to be variable, possibly due to a range of factors including several barriers facing families, training limitations for professionals, and resource constraints for health services. Further, the linkages between such programs in the community at the primary care level and the referral and clinical care pathways for those identified with developmental disorders may not be fully developed. We expect that this study will provide considerable insight into understanding the determinants of developmental risk as well as how best to engage families and professionals to identify those at risk sufficiently early to provide the best opportunities for early intervention.

Working together with policy makers, the evidence from the WMG study is expected to be used to improve the uptake of developmental surveillance by addressing the barriers from both the system and service delivery perspective, as well as from the parental awareness, attitudes and help seeking behaviours. Finally, the findings from the Diagnostic Test Accuracy component of the study will help to improve the surveillance tools and the related processes in order to minimise the adverse impact of false negative results from surveillance, and to ensure optimal outcomes for children who are identified as being at risk of a developmental disorder. The findings on the associated individual, child, and population characteristics are expected to inform ongoing planning and delivery of the NSW surveillance program. The findings will also provide the opportunity to compare the NSW model with other national and international models. Together these findings will yield an evidence base of the risk and resilience factors determining developmental disorders. The processes developed in this study for effective partnership will also have important implications for the ways in which future collaborations can be forged between those in academia, service delivery, and policy making.

## Abbreviations

AAP: American Academy of Paediatrics
ADOS: Autism diagnostic observation schedule
ASD: Autism spectrum disorder
ASQ: Ages and stages questionnaire
ASQ: SE: Ages and stages questionnaire: social-emotional
GP: General practitioner
NSW: New South Wales
PDSQ: Prospective developmental surveillance questionnaire
PEDS: Parents’ evaluation of developmental status
PHR: Personal health record
M-CHAT: Modified checklist for autism in toddlers
NBQ: Newborn baseline questionnaire
SWSLHD: South Western Sydney Local Health District
WMG: Watch me grow study.
